# Lymphocyte Kinetics and Outcomes After Comprehensive Involved-Site Radiotherapy for Oligometastases

**DOI:** 10.3390/cancers18132074

**Published:** 2026-06-26

**Authors:** Deep Patel, Megha Schmalzle, Michaela Young, Leonidas Salichos, Johnny Kao

**Affiliations:** 1 Department of Biomedical Sciences, College of Osteopathic Medicine, New York Institute of Technology, Old Westbury, NY 11568, USA; mschma01@nyit.edu (M.S.); johnny.kao@chsli.org (J.K.); 2 Department of Radiation Oncology, Good Samaritan University Hospital, West Islip, NY 11795, USA; 3 The Cancer Institute, Good Samaritan University Hospital, West Islip, NY 11795, USA; michaela.young@chsli.org; 4 Department of Biological and Chemical Sciences, New York Institute of Technology, New York, NY 10023, USA; lsalicho@nyit.edu; 5 Biomedical Data Science Center, New York Institute of Technology, Old Westbury, NY 11568, USA

**Keywords:** oligometastatic disease, lymphopenia, radiotherapy, involved-site radiotherapy, metastasis-directed therapy, overall survival

## Abstract

Purpose: Lymphopenia is a common adverse event following radiotherapy, but its prognostic relevance following comprehensive involved-site radiation (ISRT) for oligometastatic disease is unknown. We evaluated lymphocyte kinetics after ISRT for oligometastases and tested whether treatment-related lymphopenia was associated with overall survival (OS) and modified progression-free survival (mPFS). Patients and Methods: We performed a single-institution registry study of consecutive patients with 1 to 5 distant metastases treated with comprehensive ISRT by a single radiation oncologist from 2014 to 2023. Systemic therapy was administered at clinician discretion. Absolute lymphocyte count (ALC) was collected at baseline, during radiation and at 1, 3 and 12 months after radiotherapy. Lymphopenia was graded using CTCAE v5.0 (grade 1, ALC < 1000 cells/µL; grade ≥ 3, ALC < 500 cells/µL). OS and mPFS (defined as death or metastatic progression not salvageable with further local therapy) were estimated by the Kaplan–Meier method. Associations were evaluated using univariable and multivariable Cox proportional hazards models. Results: Among 177 patients, the 5-year OS was 39.6% and the median OS was 42.8 months. Median ALC declined from 1400 cells/µL at baseline to 800 cells/µL during radiotherapy (*p* < 0.01) and 700 cells/µL at a median of 0.7 months after radiation (*p* < 0.01). Partial recovery was observed at 1000 cells/µL at 3 months (*p* < 0.001) and 1000 cells/µL at 1 year (*p* < 0.01). Baseline ALC <1000 cells/µL was associated with worse OS on univariable analysis (*p* = 0.04) but not on multivariable analysis. Although 27% developed grade ≥ 3 lymphopenia within 3 months of radiation, the 5-year overall survival was 41.4% without lymphopenia versus 31.9% with lymphopenia (*p* = 0.60). On multivariable analysis, ECOG performance status (HR 1.9, *p* < 0.01), age (HR 1.04, *p* < 0.01), albumin (HR 0.6, *p* = 0.03), and pre-radiation chemotherapy (HR 3.1, *p* < 0.01) independently predicted overall survival. Conclusions: Comprehensive ISRT for oligometastatic disease was associated with a sustained decrease in median ALC. Treatment-related lymphopenia was not independently associated with OS in this heterogeneous cohort. The disease control benefit of metastasis-directed therapy may outweigh potential detrimental immunologic effects of radiation-induced lymphopenia.

## Simple Summary

Although radiation therapy is frequently utilized to treat patients with limited metastatic disease, it can reduce lymphocyte counts, which may be functionally important. At present, physicians do not know whether treatment-related lymphopenia influences survival in patients with oligometastatic cancer who receive comprehensive radiation therapy. This study examined the effects of radiation therapy on lymphocyte counts and its association with cancer progression and overall survival. We found that lymphocyte levels commonly declined after treatment and did not fully return to baseline levels. Although only hypothesis-generating, we could not demonstrate any statistically significant association between lymphopenia and worse survival outcomes following radiation. These findings add to a growing body of research on lymphopenia and may help guide future research and clinical decisions.

## 1. Introduction

Radiation therapy is an important treatment modality for the curative and palliative treatment of adult solid tumors [1]. In the setting of oligometastatic disease, metastasis-directed radiation significantly improves disease-free survival and overall survival compared to systemic therapy alone [2]. In a large cohort of consecutive patients with oligometastases treated with comprehensive involved site radiation (ISRT), the 5-year overall survival was 38% and many long-term survivors were exceptional responders without evidence of disease recurrence [3]. Despite advances in systemic therapy for stage IV cancer, durable complete remissions remain uncommon with systemic therapy alone. This highlights the importance of metastasis-directed therapy in achieving deep and durable remissions [4,5,6,7].

Circulating peripheral blood lymphocytes are highly sensitive to low doses of ionizing radiation and radiation therapy is associated with acute and late lymphopenia, an adverse effect of increasing concern in the immunotherapy era [8,9,10,11,12]. Recent meta-analyses of non-randomized data suggest that severe lymphopenia is associated with worse overall survival at 2 years, although the absolute effect size was less than 5% [13,14].

Contradicting the emerging narrative that treatment-induced lymphopenia inherently contributes to poor survival outcomes, radiation therapy for early-stage breast and prostate cancers achieves a 5-year relative survival above 99% [15]. Additionally, the randomized TRANSMET trial shows that treating colorectal cancer with liver-only metastases with liver transplantation followed by immunosuppression markedly improved overall survival compared to chemotherapy alone [16]. If metastatic colorectal patients experienced improved outcomes despite iatrogenic immunosuppression, it is possible that the outcomes of cancer patients with lymphopenia from radiotherapy may not be as dire as previous literature suggests.

The incidence, kinetics and prognostic significance of lymphopenia following comprehensive radiation for oligometastases remain poorly characterized [8]. Therefore, we investigated whether comprehensive ISRT for oligometastases impacts lymphocyte abundance and whether resultant lymphopenia is associated with survival outcomes.

## 2. Materials and Methods

### 2.1. Patient Population

This registry study was approved by the Good Samaritan University Hospital Institutional Review Board with a waiver of informed consent. The study population consisted of consecutive patients with oligometastases (defined as 1 to 5 distant metastases on whole-body imaging) referred to a single radiation oncologist between January 2014 and December 2023. All visible disease sites were targeted with local therapy. Systemic therapy was administered at the discretion of the treating medical oncologist or urologist. Treatments were categorized as cytotoxic chemotherapy, immunotherapy, hormonal therapy or biologically targeted therapy. Stereotactic radiation was utilized when technically feasible and considered safe. Locally advanced lung cancers were treated with either hypofractionated or conventionally fractionated intensity-modulated radiation therapy (IMRT). Based on physician preference, most primary prostate cancers were treated with either moderately or conventionally fractionated radiation instead of stereotactic body radiotherapy to reduce the risk of late grade ≥ 2 genitourinary toxicity [17,18]. Biological equivalent dose (BED) was calculated using the linear-quadratic model with an α/β ratio of 10.

Prognostically relevant clinical variables were selected based on previously published studies of patients with distant metastases treated with radiation [7,19,20,21]. The variables include ECOG performance status, gender, age, primary tumor site, metastasis sites, bone-only metastases, extent of prior systemic therapy regimens for distant metastases, hospitalization during the prior 3 months, pretreatment albumin, baseline neutrophil-to-lymphocyte ratio, radiation dose and volume, systemic therapy administered before, during and after radiation and EORTC oligometastasis disease classification. Patients underwent regular blood draws as standard of care. Absolute lymphocyte count (ALC) was recorded prior to radiation for oligometastases, during radiation and at 1, 3 and 12 months after radiation. We recorded systemic therapy administered at each ALC timepoint, including before radiation, during radiation and throughout the first year following radiation. For patients requiring multiple courses of radiation for comprehensive treatment separated by several months (e.g., lung cancer with brain metastases), ALC values for the first four timepoints were attributed to the first radiotherapy course. For the late timepoint, lymphocyte count was recorded at 1 year after the most recent radiotherapy course. The Common Terminology Criteria for Adverse Events version 5.0 defines grade 1 lymphopenia as <1000 cells/µL and grade 3 lymphopenia as <500 cells/µL [13].

### 2.2. Statistical Analysis

Follow-up length was defined as the initial consultation for oligometastases to most recent follow-up or death. Modified progression-free survival was defined as time to death or progression that could not be salvaged by further local therapy [22]. Progression was considered salvageable when the treating physician believed it could still be reasonably managed with additional local therapy based on the extent of disease, feasibility of treatment, and the overall clinical picture. Differences between baseline ALC and post-radiation ALC were compared using the Wilcoxon signed-rank test for paired data. ALC analyses at each timepoint were based on the patients who had laboratory data available. To address the potential risk of immortal time bias, landmark analyses were performed at 1, 3, and 12 months. Patients who died or were censored before the landmark were excluded, and follow-up was restarted from the landmark timepoint. Clinical and treatment-related predictors of grade ≥ 3 lymphopenia were evaluated using univariable and multivariable logistic regression. Overall survival (OS) and modified progression-free survival (mPFS) were estimated using the Kaplan–Meier method, with differences between groups assessed using the log-rank test. Exploratory subgroup analyses by immunotherapy exposure and primary tumor type were performed using log-rank tests. To identify independent prognostic factors for OS and mPFS, univariable Cox proportional hazards regression was performed for each covariate.

Variables with a *p* value of <0.1 on univariable Cox analysis, along with lymphopenia, were entered into multivariable Cox models. The primary multivariable Cox models were performed as complete-case analyses rather than imputing missing data. The proportional hazards assumption was assessed using Schoenfeld residuals. To avoid overfitting, backward removal was performed for variables with a *p* value >0.1. All statistical tests were two-sided with a significance level of 0.05. Analyses were performed in Stata 13.1 (StataCorp LLC, College Station, TX, USA).

## 3. Results

### 3.1. Patient and Treatment Characteristics

There were 177 patients with oligometastases with a median follow-up of 44.0 months among surviving patients. The 5-year overall survival was 39.6% [95% CI 31.2% to 47.9%] with a median survival of 42.8 months [95% CI 29.8 to 53.7 months]. Patient and treatment characteristics are summarized in Table 1. The median BED10 to the primary tumor and nodes was 73.4 Gy (IQR 59.5 to 79.2 Gy) and 60.0 Gy (IQR 50.7 to 75.0 Gy) to distant metastases. The median gross tumor volume (GTV) was 48.6 cm^3^ (IQR 14.9 to 120.2 cm^3^).

### 3.2. Impact of Radiation on Absolute Lymphocyte Counts

**Figure 1:**
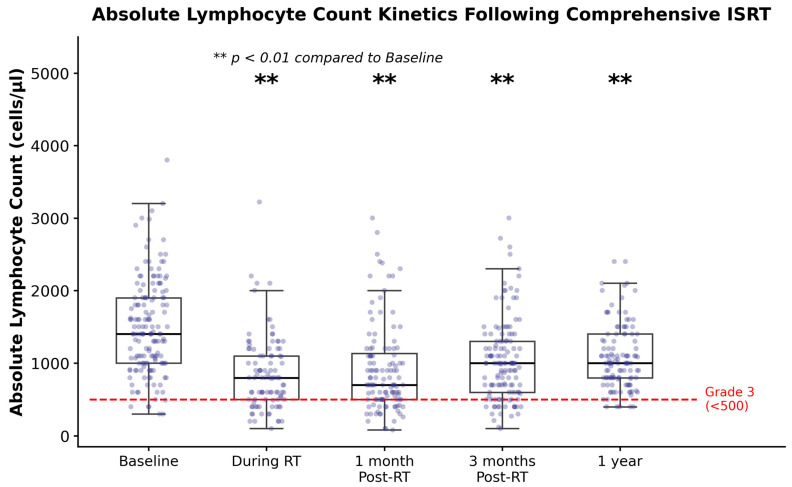
Absolute lymphocyte count (ALC) kinetics before, during and after comprehensive involved-site radiotherapy. The data is represented as a box-and-whisker plot demonstrating median and interquartile range with underlying individual patient distribution. The dashed red line indicates the cutoff for grade 3 lymphopenia (<500 cells/µL). ** *p* < 0.01 compared to baseline ALC.

The median pre-radiation absolute lymphocyte count was 1400 cells/µL (IQR 952 to 1900) (Figure 1). ALC data were available for 155 patients at baseline, 97 during radiotherapy, 120 at 1 month post-radiotherapy, 134 at 3 months and 116 at 1 year. During radiotherapy, the median ALC was 800 cells/µL (IQR 490 to 1190; *p* < 0.001). At a median time of 0.7 months post-radiotherapy, the median ALC was 700 cells/µL (IQR 426 to 1112; *p* < 0.001). At a median follow-up of 3.0 months after radiation, the median ALC was 1000 cells/µL (IQR 581 to 1310; *p* < 0.001). At a median follow-up of 1.0 years after radiation, the median ALC was 1000 cells/µL (IQR 790 to 1400, *p* = 0.002) and remained below baseline. The percentage of patients with an ALC <500 cells/µL was 4% at baseline and 4% at 1-year follow-up.

Grade ≥ 3 lymphopenia within 3 months of radiation occurred in 27% of patients. On multivariable logistic regression, baseline lymphopenia (OR 5.4, *p* < 0.01) and post-radiation chemotherapy (OR 3.6, *p* < 0.01) were the only independent predictors of post-treatment grade ≥ 3 lymphopenia.

### 3.3. Survival Outcomes

**Figure 2:**
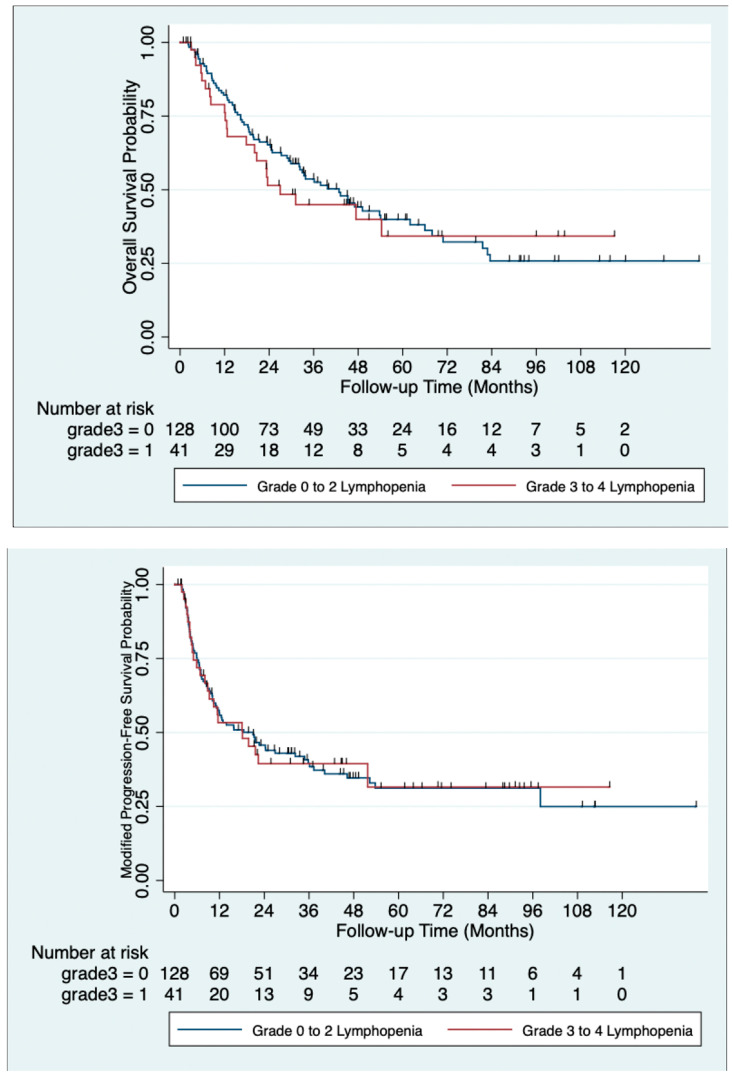
(**a**) Overall survival probability stratified by low-grade (0–2) versus high-grade (3–4) lymphopenia. (**b**) Progression-free survival probability stratified by low-grade (0–2) versus high-grade (3–4) lymphopenia.

Among the lymphopenia metrics evaluated, only baseline ALC < 1000 cells/µL was associated with worse overall survival on univariable analysis (*p* = 0.04) (Table 2). Additional survival analyses at later timepoints are shown in Supplementary Table S1, with patients grouped by lymphopenia grade at each timepoint. Since post-treatment ALC measurements were only available in patients who survived long enough to have follow-up laboratory testing, these analyses should be interpreted as exploratory. Separate landmark analyses at 1, 3, and 12 months did not show a significant OS difference by grade ≥ 3 lymphopenia status (Supplementary Table S2). The 5-year overall survival was 41.4% without lymphopenia versus 31.9% for patients with grade ≥ 3 lymphopenia within 3 months of radiation (*p* = 0.60) (Figure 2a).

Other univariable predictors of overall survival include ECOG performance status (*p* < 0.01), primary tumor type (*p* < 0.01), liver metastases (*p* < 0.01), baseline serum albumin (<0.01), pre-radiation chemotherapy (*p* = 0.03) and post-radiation non-cytotoxic systemic therapy (*p* = 0.04). A stratified multivariable Cox proportional hazards model was performed to identify independent predictors of overall survival. To account for varying time-dependent hazard functions, the model was stratified by primary tumor site. ECOG performance status (HR 1.9, 95% CI [1.3 to 2.8], *p* < 0.01), age (HR 1.04, 95% CI [1.02 to 1.07], *p* < 0.01), albumin (HR 0.6, 95% CI [0.4 to 1.0], *p* = 0.03), and pre-radiation chemotherapy (HR 3.1, 95% CI [1.8 to 5.4], *p* < 0.01) independently predicted overall survival (Table 3). In the final model, post-radiation grade ≥ 3 lymphopenia (HR 1.4, 95% CI [0.8 to 2.4], *p* = 0.22) was not an independent predictor of survival.

The 5-year modified PFS was 30.5% without lymphopenia versus 30.7% for patients with grade ≥ 3 lymphopenia within 3 months of radiation (*p* = 1.00) (Figure 2b). For modified progression-free survival, ECOG performance status (HR 1.6, 95% CI [1.2 to 2.1], *p* < 0.01), age (HR 1.02, 95% CI [1.00 to 1.04], *p* = 0.05), pre-radiation chemotherapy (HR 3.2, 95% CI [2.0 to 5.3], *p* < 0.01) and post-radiation non-cytotoxic systemic therapy (HR 0.3, 95% CI [0.2 to 0.7], *p* < 0.01) remained significant on multivariable analysis. To address possible confounding from pre-radiation cytotoxic chemotherapy, we performed a sensitivity analysis excluding those patients. In that group, grade ≥ 3 lymphopenia still was not significantly associated with OS or mPFS on Kaplan–Meier analysis (*p* = 0.712 and *p* = 0.330, respectively) or adjusted Cox analysis (OS: HR 0.85, 95% CI 0.30–2.46, *p* = 0.767; mPFS: HR 0.65, 95% CI 0.23–1.86, *p* = 0.422) (Supplementary Table S3).

### 3.4. Exploratory Subgroup Analyses

Exploratory subgroup analyses were performed by immunotherapy exposure and primary tumor type. Grade ≥ 3 lymphopenia was not associated with significant differences in OS or mPFS within immunotherapy subgroups or within lung, prostate, breast, and other primary tumor groups (Supplementary Table S4). These analyses should be interpreted cautiously because several subgroups had small numbers of events.

## 4. Discussion

In this retrospective tumor-agnostic cohort of patients with oligometastatic disease treated with comprehensive involved-site radiotherapy, we did not observe a significant association between lymphopenia and overall survival or disease progression. Most of the prior studies were retrospective and the reported multivariable analyses often did not include key prognostic variables such as albumin, liver metastases, performance status and tumor burden, thus limiting causal inference [8,11,13,23,24]. It is also possible that studies demonstrating no association between lymphopenia and survival were less likely to complete the publication process [25].

This study confirms prior reports demonstrating that radiation-associated lymphopenia is common and does not completely return to baseline at one year [23]. Key predictors of radiation-induced lymphocyte depletion include treatment site, target volume, dose per fraction, lymphocyte-rich organ and circulating blood pools, concurrent chemotherapy, baseline lymphopenia and age [14,23,26,27]. In this tumor-site agnostic cohort, multiple metastases, non-stereotactic technique, chemotherapy administration and baseline lymphopenia predicted subsequent lymphopenia. In a novel finding, pre-radiation chemotherapy was associated with a 3-fold-increased risk of progression or death (*p* < 0.01). Although confirmatory data for oligometastases are not available, recent data in non-Hodgkin’s lymphoma demonstrated an excess incidence of non-lymphoma deaths from cytotoxic chemotherapy but not involved-site radiotherapy [28]. Taken together, these findings raise the possibility that some associations previously attributed to radiotherapy may also be influenced by prior or concurrent chemotherapy.

The principle that the benefits of disease eradication outweigh the potential risks of iatrogenic lymphopenia is consistent with a large body of randomized data in non-metastatic solid tumors. For example, regional nodal irradiation reduces the risk of distant metastasis and breast cancer death in node-positive breast cancer despite substantially increasing lymphocyte irradiation compared to breast irradiation alone [29,30]. In high-risk non-metastatic prostate cancer, adding pelvic radiation to hormonal therapy improved overall survival compared to hormonal therapy alone [31]. Outcomes have also improved for locally advanced lung and head and neck cancers treated with combined radiation and lymphocyte-depleting chemotherapy despite irradiation of involved or even elective lymph nodes [32,33].

For metastatic solid tumors, the randomized TRANSMET trial demonstrated that ablating gross unresectable liver metastases from colorectal cancer through liver resection and transplantation markedly improved overall survival despite requiring long-term immunosuppression [16]. These findings are concordant with the SABR-COMET trial that demonstrated improved overall survival with consolidative radiation for oligometastases despite the well-established lymphopenia-inducing effect of metastasis-directed radiation [4]. More recently, focal and comprehensive radiotherapy approaches for polymetastatic disease have reported promising oncologic outcomes, including improved overall survival [34,35,36]. By contrast, treating with low-volume and reduced-dose-intensity palliative radiation for polymetastasis intended to avoid interfering with primary systemic therapy was associated with an exceptional response rate of only 1% [7].

In this study, the prognostic impact of radiation-induced lymphopenia appears to be context-specific rather than uniformly detrimental. On multivariable analysis, only baseline lymphopenia and prior chemotherapy predicted severe lymphopenia within 3 months of treatment, suggesting that baseline patient factors may be more important than radiation technique or dose intensity in this cohort. Although lymphopenia following radiation for oligometastatic disease was common, acute grade ≥ 3 lymphopenia occurred in 27% of patients but only 4% had persistent grade ≥ 3 lymphopenia at 1 year. Although hypothesis-generating, these findings suggest that a post-treatment numerical decline in ALC was not clearly associated with worse survival outcomes.

Immunotherapy is increasingly used in patients with stage IV cancer [37]. In this cohort of patients with oligometastases treated from 2014 to 2023, 25% of patients received immunotherapy with a median survival of 24.6 months versus 43.9 months without immunotherapy (*p* = 0.12). Although incompletely understood, the timing of radiation and immunotherapy likely impacts immunotherapy efficacy [32,38]. In this study, neither any grade lymphopenia (*p* = 0.68) nor high-grade lymphopenia (*p* = 0.41) following radiation was associated with worse survival among patients treated with immunotherapy. However, this study included fewer patients receiving immunotherapy compared to the larger Dana Farber experience that demonstrated that performance status, albumin, and pre-immunotherapy ALC < 500 cells/µL were important prognostic factors on multivariable analysis [10]. The interaction between radiation-induced lymphopenia and immunotherapy response is being extensively investigated through efforts such as the ImmunoRad ROBIN consortium. For the majority of oligometastatic patients not receiving immunotherapy, treatment-related lymphopenia was not associated with worse median or long-term survival.

Reducing radiation-induced lymphopenia remains a worthwhile goal, particularly for patients receiving immunotherapy. Proton beam radiotherapy is relatively lymphocyte-sparing compared to photon therapy and combined recent research on secondary lymphoid organs may enable more robust lymphocyte-sparing radiotherapy [14,39]. Pharmacologic approaches to accelerating lymphocyte recovery, including the IL-15 superagonist N-803, are under active investigation [40]. While these strategies are based on the concern that iatrogenic lymphopenia may be harmful, this study shows that the association between baseline lymphopenia and worse overall survival on univariable analysis disappeared after adjustment for prior chemotherapy, age, performance status and low albumin. Preserving lymphocyte counts may also improve survivorship by reducing infection-related complications [23,41]. Prospective trials with adequate adjustment for confounders are needed to determine whether mitigating treatment-induced lymphopenia improves cancer-related outcomes.

This study has important limitations. It represents a single-physician retrospective cohort study with a heterogeneous, tumor-agnostic population seen in real-world community oncology practice. Since the tumor types in this cohort represent diverse clinical behavior and prognosis, the pooled analyses are harder to interpret, and the findings may not apply equally across all disease settings. Given the heterogeneity of the cohort and the retrospective design, the absence of a statistically significant association should not be interpreted as definitive evidence that treatment-related lymphopenia has no prognostic impact. Indeed, the confidence intervals for this retrospective study cannot rule out a moderate detrimental effect. Lymphopenia and survival can be impacted by systemic treatment intensity, disease burden, frailty, or baseline condition. Our multivariable models were limited to patients with complete covariate data, which reduced the sample size and may have introduced bias if the excluded patients differed systematically from those included in the final models. Although we had information on treatment site, radiation technique, BED, and GTV, we did not have more detailed organ-specific dosimetric data for relevant structures such as the lung, heart, spleen, bone marrow, and circulating blood pool, which makes the biologic interpretation of the lymphopenia findings more limited [27]. Finally, the later post-treatment ALC analyses are vulnerable to survivor bias, because only patients who remained alive and had follow-up laboratory testing could be included at those timepoints. ALC is an inexpensive but crude biomarker and more sophisticated immune profiling through flow cytometry, single-cell RNA-seq or spatial transcriptomics would be illuminating. These results should be considered hypothesis-generating and require prospective validation through collaborative efforts such as the E^2^-RADIatE OligoCare consortium [42].

## 5. Conclusions

Comprehensive ISRT is an active regimen for oligometastases with potential for long-term remissions despite frequent mostly low-grade lymphopenia. Treatment-related lymphopenia was not independently associated with overall survival or modified progression-free survival in this heterogeneous cohort. Although these findings should be interpreted with caution, they provide useful context for future studies and may help guide clinical decision-making.

## Figures and Tables

**Table 1:** Kaplan–Meier estimates of overall survival and progression-free survival for oligometastatic patients (*n* = 177), stratified by baseline clinical characteristics. Median survival times (in months) and 5-year survival percentages are shown. *p*-values represent log-rank tests comparing survival curves between groups.

	Number	Percent (%)	Median Overall Survival (Months)	5-Year Overall Survival	*p* Value	Median Progression-Free Survival (Months)	5-Year Progression-Free Survival	*p* Value
Total	177	100	42.8	39.6%		19.8	31.9%	
ECOG Performance Status					<0.01 *			<0.01 *
0–1	129	73	45.2	42.2%		21.5	34.2%	
2–4	48	28	29.8	34.8%		9.6	26.7%	
Gender					0.13			0.27
Male	98	55	49.2	45.3%		26.9	32.4%	
Female	79	45	28.9	32.1%		11.6	31.7%	
Age					<0.01 *			<0.01 *
<70	85	48	48.8	44.6%		18.1	36.3%	
≥70	92	52	37.9	35.0%		21.0	28.0%	
Primary site					<0.01			<0.01
Lung	60	34	28.9	34.7%	0.51	12.6	25.9%	0.44
Prostate	28	16	81.5	64.0%	<0.01	52.3	47.4%	<0.01
Breast	19	11	82.9	55.7%	0.06	34.8	47.6%	0.08
Other	70	40	24.6	28.2%	ref	10.4	26.2%	ref
Metastasis site								
Bone	61	34	43.9	41.4%	0.29	24.3	29.8%	0.31
Brain	47	27	24.8	29.6%	0.27	10.1	31.5%	0.29
Distant Lymph Nodes	36	20	49.2	47.3%	0.27	40.2	38.1%	0.11
Lung	34	19	42.8	35.8%	0.87	21.5	29.8%	0.52
Liver	16	9	10.4	29.2%	<0.01	10.4	11.4%	0.03
Prior Palliative Chemotherapy Regimens					0.20			0.18
0–1	146	82	45.2	40.9%		21.5	33.5%	
≥2	31	18	29.8	32.9%		13.8	24.1%	
Hospitalized During Prior 3 Months					0.51			0.23
No	114	64	43.9	40.7%		21.6	33.3%	
Yes	63	36	31.7	37.2%		10.1	28.9%	
Baseline Albumin					<0.01 *			<0.01 *
≥3.8 g/dL	78	44	45.3	50.3%		46.2	45.3%	
<3.8 g/dL	84	47	14.9	28.3%		10.1	16.1%	
Missing	15	8	49.2	38.6%		37.3	43.8%	
Oligometastatic State					0.12			0.21
De novo	138	78	45.2	40.0%	ref	21.6	31.8%	ref
Induced	24	14	23.6	24.2%	0.07	10.9	17.8%	0.08
Repeat	15	8	NR	53.9%	0.41	10.4	46.7%	0.68
Number of Active Metastases					0.20			0.13
0 to 3	161	90	37.9	38.6%		18.1	30.0%	
4 to 5	16	9	47.4	45.7%		NR	59.3%	
RT Technique					0.33			0.51
Stereotactic only	44	25	33.4	30.9%	ref	11.9	28.5%	ref
IMRT only	73	41	49.2	47.0%	0.16	21.5	35.6%	0.24
SRT and IMRT	40	23	33.8	40.2%	0.84	21.5	23.8%	0.59
Pre-RT Systemic Therapy					<0.01			<0.01
None	114	65	54.1	46.9%	ref	32.3	37.7%	ref
Non-Cytotoxic	19	11	37.9	39.8%	0.68	21.0	32.1%	0.35
Cytotoxic	43	24	19.9	18.3%	<0.01	9.6	15.7%	<0.01
Post-RT Systemic Therapy					0.02			<0.01
None	38	22	18.6	40.2%	ref	7.2	25.0%	
Non-Cytotoxic	75	43	62.0	52.8%	0.04	52.3	46.7%	<0.01
Cytotoxic	62	35	28.9	25.8%	0.86	10.9	20.1%	0.80

* Analyzed as a continuous variable.

**Table 2:** Kaplan–Meier estimates of overall survival and progression-free survival for oligometastatic patients who underwent radiotherapy, stratified by absolute lymphocyte count (ALC) at certain time points and the presence of lymphopenia. ALC was collected before, during, and after radiation at 1, 3, and 12 months. Median survival times (months) and 5-year survival rates are shown. *p*-values represent log-rank tests comparing survival curves between groups.

	Percent (%)	Median Overall Survival (Months)	5-Year Overall Survival	*p* Value	Median Progression-Free Survival (Months)	5-Year Progression-Free Survival	*p* Value
Total *		42.8	39.6%		19.8	31.9%	
Baseline lymphocytes ≥ 1000	79	45.2	42.7%		21.0	33.1%	
Baseline lymphocytes < 1000	21	23.3	21.1%	0.04	10.4	19.8%	0.13
Baseline lymphocytes < 500	4	33.4	26.7%	0.76	10.4	30.0%	0.82
During RT lymphocytes ≥ 1000	35	28.9	41.8%		15.8	29.6%	
During RT lymphocytes < 1000	65	47.4	35.4%	0.54	22.4	26.6%	0.55
During RT lymphocytes < 500	23	47.1	31.6%	0.77	19.8	20.8%	0.84
1 Month after RT lymphocytes ≥ 1000	34	29.3	28.9%		10.1	26.8%	
1 Month after RT lymphocytes < 1000	66	32.1	35.3%	0.75	18.1	29.0%	0.54
1 Month after RT lymphocytes < 500	20	23.6	29.5%	0.78	18.1	33.5%	0.70
3 Months after RT lymphocytes ≥ 1000	53	43.2	36.8%		13.8	29.6%	
3 Months after RT lymphocytes < 1000	47	27.0	41.6%	0.61	18.1	35.6%	0.67
3 Months after RT lymphocytes < 500	14	23.3	21.6%	0.35	18.1	36.1%	0.70
1 Year after RT lymphocytes ≥ 1000	57	70.9	50.7%		51.7	47.7%	
1 Year after RT lymphocytes < 1000	43	54.3	49.4%	0.73	46.2	34.6%	0.69
1 Year after RT lymphocytes < 500	4	47.4	30.0%	0.59	21.6	40.0%	0.86
Any Grade ≥ 3 Lymphopenia within 3 months				0.60			1.00
Yes	27	27.0	31.9%		18.5	30.5%	
No	73	43.2	41.4%		18.0	30.7%	

* Median OS = 42.8 months; 5-year OS = 39.6%.

**Table 3:** Multivariable Cox proportional hazards estimates of overall survival and modified progression-free survival for oligometastatic patients with complete covariate data *(n* = 146). Hazard ratios (HRs), 95% confidence intervals (CIs), and *p*-values are shown.

	Overall Survival Hazard Ratio (95% CI)	OS *p* Value	Modified Progression-Free Survival Hazard Ratio (95% CI)	Modified Progression-Free Survival *p* Value
ECOG Performance Status	1.92 (1.33–2.77)	<0.001 *	1.58 (1.16–2.14)	0.004 *
Age	1.04 (1.02–1.07)	0.001 *	1.02 (1.00–1.05)	0.045 *
Albumin	0.63 (0.41–0.96)	0.033 *	0.75 (0.51–1.09)	0.134 *
Pre-RT Systemic Therapy				<0.001
Non-Cytotoxic vs. None	1.58 (0.62–4.00)	0.336	1.77 (0.81–3.88)	0.154
Cytotoxic vs. None	3.12 (1.80–5.42)	<0.001	3.25 (2.00–5.28)	<0.001
Post-RT Systemic Therapy		0.025		<0.001
Non-Cytotoxic vs. None	0.50 (0.24–1.02)	0.056	0.35 (0.18–0.66)	0.001
Cytotoxic vs. None	1.12 (0.57–2.21)	0.736	0.89 (0.48–1.63)	0.696
Grade ≥ 3 Early Lymphopenia	1.40 (0.81–2.41)	0.223	1.09 (0.66–1.80)	0.737

* Analyzed as a continuous variable.

## Data Availability

The data presented in this study are available on request from the corresponding author. The data are not publicly available due to privacy or ethical restrictions.

## Supplementary Materials

The following supporting information can be downloaded at https://www.mdpi.com/article/10.3390/cancers18132074/s1. Table S1: Complete Survival Summary by Absolute Lymphocyte Count at All Recorded Clinical Timepoints; Table S2: Landmark analyses of overall survival by grade ≥ 3 lymphopenia status; Table S3: Sensitivity analyses excluding patients who received pre-radiation cytotoxic chemotherapy; Table S4: Subgroup analyses by immunotherapy exposure and primary tumor type.
