# Supplementary material for: Lymphocyte Kinetics and Outcomes After Comprehensive Involved-Site Radiotherapy for Oligometastases
Source: Cancers (Basel). 2026 Jun 26;18(13):2074. doi: 10.3390/cancers18132074 (PMC13359456; doi:10.3390/cancers18132074)
Supplement: Supplementary file 1 [file cancers-18-02074-s001.zip › cancers-4330250-supplementary.pdf]

Supplementary Table S1. Complete Survival Summary by Absolute Lymphocyte Count at All Recorded Clinical Timepoints

Statistical note: For the later follow-up timepoints, patients were grouped by absolute lymphocyte count (ALC) into separate CTCAE-based categories: grade 0–1 (≥1000 cells/µL), grade 2 (500–999 cells/µL), and grade 3–4 (<500 cells/µL). Each patient was included in only one category at each timepoint. "During RT" refers to measurements obtained during radiotherapy, "Days After RT" refers to the early post-radiotherapy assessment, "Months After RT" refers to the 3-month follow-up assessment, and "1 Year After RT" refers to the follow-up assessment approximately 1 year after the most recent radiotherapy course.

**Variable**	**N (%)**	**Median OS (Months)**	**5-Year OS (%)**	**P-value**	**Median mPFS (Months)**	**5-Year mPFS (%)**	**P-value**
**During RT (Acute) ALC**				**0.71**			**0.76**
- Grade 0-1 (>= 1000)	34 (35%)	28.8	41.8%		15.8	29.6%	
- Grade 2 (500-999)	41 (42%)	47.4	37.7%		24.3	29.1%	
- Grade 3-4 (< 500)	22 (23%)	47.1	31.6%		19.8	20.8%	
**Days After RT ALC**				**0.87**			**0.82**
- Grade 0-1 (>= 1000)	41 (34%)	29.3	28.9%		10.1	26.8%	
- Grade 2 (500-999)	55 (46%)	45.2	37.7%		21.0	26.5%	
- Grade 3-4 (< 500)	24 (20%)	23.6	29.5%		18.1	33.5%	
**Months After RT ALC**				**0.64**			**0.76**

- Grade 0-1 (>= 1000)	71 (53%)	43.2	36.8%		13.8	29.6%	
- Grade 2 (500-999)	44 (33%)	31.1	45.5%		18.5	36.6%	
- Grade 3-4 (< 500)	19 (14%)	23.3	21.6%		18.1	36.1%	
**1 Year After RT ALC**				**0.85**			**0.92**
- Grade 0-1 (>= 1000)	66 (57%)	70.9	50.7%		51.7	47.7%	
- Grade 2 (500-999)	45 (39%)	62.0	51.7%		46.2	33.6%	
- Grade 3-4 (< 500)	5 (4%)	47.4	30.0%		21.6	40.0%	

Supplementary Table S2. Landmark analyses of overall survival by grade ≥3 lymphopenia status

Statistical note: Landmark analyses were performed at 1, 3, and 12 months. Patients who expired or were censored before each landmark were excluded, and follow-up was restarted from the landmark timepoint. Overall survival was compared between patients with and without grade ≥3 lymphopenia at each landmark using the log-rank test.

Landmark timepoint	No grade ≥3 lymphopenia, n	Grade ≥3 lymphopenia, n	Events in no grade ≥3 group, n	Events in grade ≥3 group, n	Log-rank p-value
1 month	96	24	57	15	**0.78**
3 months	114	19	63	11	**0.32**
12 months	107	5	47	3	**0.52**

Supplementary Table S3. Sensitivity analyses excluding patients who received pre-radiation cytotoxic chemotherapy

Statistical note: Adjusted Cox models included grade ≥3 lymphopenia, ECOG performance status, age, and albumin. Patients who received pre-radiation cytotoxic chemotherapy were excluded from all analyses in this table.

Outcome	Analysis	Grade ≥3 lymphopenia estimate	95% CI	P-value
Overall Survival	Kaplan–Meier			**0.71**
Overall Survival	Adjusted Cox model	HR 0.85	0.30–2.46	**0.77**
Modified progression-free survival	Kaplan–Meier			**0.33**
Modified progression-free survival	Adjusted Cox model	HR 0.65	0.23–1.86	**0.42**

Supplementary Table S4. Subgroup analyses by immunotherapy exposure and primary tumor type

Statistical note: N represents the total number of patients in each subgroup. Subgroup analyses were performed using log-rank tests comparing patients with and without grade ≥3 lymphopenia within each subgroup. These analyses were exploratory and should be interpreted cautiously because several subgroups had a small number of events.

Subgroup	N	OS log-rank p-value	mPFS log-rank p-value
Received immunotherapy	45	**0.41**	**0.23**
Did not receive immunotherapy	132	**0.94**	**0.50**
Lung	60	**0.84**	**0.40**
Prostate	28	**0.61**	**0.86**
Breast	19	**0.41**	**0.53**
Other	70	**0.92**	**0.38**
